# Supplementary material for: Reinforcement learning of adaptive control strategies
Source: Commun Psychol. 2024 Jan 12;2:8. doi: 10.1038/s44271-024-00055-y (PMC11332247; doi:10.1038/s44271-024-00055-y)
Supplement: Supplementary file 1 — Supplementary Material [file 44271_2024_55_MOESM1_ESM.pdf]

Supplementary Table 1

Preregistered accuracy model from pilot experiment

	**Estimate**	**Est. Error**	**95% CI**
Intercept	2.063	0.081	1.905–2.220
Group	-0.048	0.081	-0.205–0.111
Congruency	0.266	0.033	0.202–0.330
Transition	0.144	0.021	0.103–0.186
Group*Congruency	0.003	0.032	-0.061–0.066
Group*Transition	-0.034	0.020	-0.074–0.005
Congruency*Transition	-0.005	0.021	-0.046–0.036
Group*Congruency*Transition	-0.027	0.019	-0.065–0.011

Supplementary Table 2

Preregistered RT model from pilot experiment

	**Estimate**	**Est. Error**	**95% CI**
Intercept	6.964	0.028	6.908–7.018
Group	-0.021	0.024	-0.068–0.027
Congruency	-0.016	0.002	-0.020–-0.011
Transition	-0.077	0.004	-0.085–-0.068
Group*Congruency	0.003	0.002	-0.001–0.008
Group*Transition	-0.000	0.004	-0.008–0.008
Congruency*Transition	0.001	0.002	-0.003–0.005
Group*Congruency*Transition	-0.000	0.002	-0.004–0.003

Supplementary Table 3

Preregistered accuracy model from Experiment 1

	**Estimate**	**Est. Error**	**95% CI**
Intercept	2.489	0.096	2.303–2.676
Group	0.020	0.093	-0.163–0.206
Congruency	0.349	0.033	0.285–0.414
Transition	0.146	0.026	0.094–0.197
Block number	0.267	0.037	0.195–0.339
Task	0.206	0.033	0.141–0.272
Group*Congruency	0.033	0.030	-0.027–0.093
Group*Transition	0.017	0.024	-0.032–0.064
Congruency*Transition	-0.004	0.023	-0.049–0.041
Group*Block number	0.019	0.035	-0.050–0.088
Congruency*Block number	-0.020	0.026	-0.071–0.031
Transition*Block number	-0.035	0.023	-0.080–0.010
Group*Congruency*Transition	-0.022	0.022	-0.065–0.021
Group*Congruency*Block number	0.009	0.024	-0.038–0.056
Group*Transition*Block number	-0.025	0.022	-0.068–0.019
Congruency*Transition*Block number	0.034	0.023	-0.012–0.080
Group*Congruency*Transition*Block number	-0.007	0.022	-0.051–0.037

Supplementary Table 4

Preregistered RT model from Experiment 1

	**Estimate**	**Est. Error**	**95% CI**
Intercept	6.750	0.029	6.693–6.806
Group	-0.007	0.027	-0.059–0.045
Congruency	-0.020	0.002	-0.025–-0.016
Transition	-0.073	0.004	-0.081–-0.065
Block number	-0.024	0.007	-0.037–-0.0118
Task	-0.036	0.005	-0.045–-0.027
Group*Congruency	-0.002	0.002	-0.006–0.003
Group*Transition	-0.003	0.004	-0.010–0.005
Congruency*Transition	0.000	0.002	-0.004–0.004
Group*Block number	0.004	0.006	-0.008–0.017
Congruency*Block number	-0.002	0.002	-0.005–0.002
Transition*Block number	0.008	0.002	0.004–0.011
Group*Congruency*Transition	-0.000	0.002	-0.004–0.003
Group*Congruency*Block number	-0.001	0.002	-0.004–0.003
Group*Transition*Block number	0.002	0.002	-0.002–0.005
Congruency*Transition*Block number	0.002	0.002	-0.003–0.006
Group*Congruency*Transition*Block number	-0.000	0.002	-0.005–0.004

Supplementary Table 5

Preregistered accuracy model from Experiment 2 (first half)

	Estimate	Est. Error	95% CI
Intercept	2.643	0.060	2.526–2.761
Group	-0.027	0.051	-0.128–0.074
Congruency	0.268	0.021	0.226–0.310
Transition	0.163	0.019	0.126–0.200
Block number	0.175	0.026	0.125–0.225
Task	0.283	0.035	0.215–0.353
Group*Congruency	0.019	0.020	-0.021–0.059
Group*Transition	-0.019	0.018	-0.054–0.016
Congruency*Transition	-0.013	0.018	-0.048–0.022
Group*Block number	-0.038	0.025	-0.087–0.011
Congruency*Block number	-0.003	0.018	-0.038–0.032
Transition1:Block number	-0.021	0.017	-0.055–0.013
Group*Congruency*Transition	0.005	0.016	-0.026–0.037
Group*Congruency*Block number	-0.049	0.017	-0.081–-0.016
Group*Transition*Block number	-0.009	0.017	-0.041–0.024
Congruency*Transition*Block number	0.019	0.017	-0.015–0.053
Group*Congruency*Transition*Block number	-0.012	0.017	-0.044–0.021

Supplementary Table 6

Preregistered RT model from Experiment 2 (first half)

	Estimate	Est. Error	95% CI
Intercept	6.759	0.017	6.726–6.791
Group	-0.001	0.016	-0.032–0.029
Congruency	-0.015	0.002	-0.018–-0.012
Transition	-0.082	0.003	-0.088–-0.077
Block number	-0.052	0.005	-0.061–-0.043
Task	-0.064	0.005	-0.074–-0.055
Group*Congruency	-0.001	0.002	-0.004–0.002
Group*Transition	0.003	0.003	-0.003–0.009
Congruency*Transition	0.001	0.001	-0.002–0.004
Group*Block number	0.002	0.005	-0.007–0.011
Congruency*Block number	-0.002	0.001	-0.005–0.001
Transition1:Block number	0.009	0.002	0.006–0.012
Group*Congruency*Transition	-0.001	0.001	-0.004–0.001
Group*Congruency*Block number	-0.002	0.001	-0.004–0.001
Group*Transition*Block number	-0.000	0.002	-0.003–0.003
Congruency*Transition*Block number	-0.002	0.001	-0.005–0.000
Group*Congruency*Transition*Block number	-0.000	0.001	-0.003–0.002

Supplementary Methods and Discussion 1

The contingency awareness model served to test whether awareness of one's reward condition (in those participants that selected either reward condition), impacted their reinforcement learning. These models neither revealed any main effect of or interaction with Awareness (with arbitrary or wrong guesses coded 0) on accuracy, nor on reaction times.

$$Accuracy/RT \sim Group * Congruency * (Transition + Task + Awareness) + (1 + Congruency * (Transition + Task + Awareness) \mid Subject) + (1 + Task \mid Stimulus)$$

Supplementary Methods and Discussion 2

We also ran models including BISBAS scores[1]. Due to convergence problems in the RT model, we ran separate models per variables of interest, namely Reward Responsiveness and BAS drive and further removed all random slopes.

$$Accuracy \sim Group * Congruency * (Transition + BAS\ Drive + BAS\ Fun + BAS\ Reward\ Responsiveness + BIS + Experiment + Task) + (1 + Congruency * (Transition + Task) \mid Subject) + (1 + Task \mid Stimulus)$$

$$RT \sim Group * Congruency * (Transition + BAS\ Reward\ Responsiveness / \ Bas\ Drive + Experiment + Task) + (1 \mid Subject) + (1 \mid Stimulus)$$

The accuracy model revealed a significant effect of BAS drive (Est. = 0.102, 95% CI [0.001, 0.204]), and BIS (Est= -0.102, 95% CI [-0.186, -0.016]), with people scoring higher on BAS showing higher accuracy and people with stronger BIS showing lower accuracy than the grand mean. We also found an interaction between group and BAS drive (Est. = -0.104, 95% CI [-0.204, -0.003]), and Group and BIS (-0.113, 95% CI [-0.196, -0.030]), with higher BAS scores being associated with higher accuracy in the group rewarded more on incongruent trials and higher scores on BIS significantly being associated with lower accuracy in the group rewarded more on congruent trials. Lastly we found an interaction between Congruency and BAS drive (Est.= 0.038, 95% CI [0.001, 0.075]) with higher scores on BAS drive being significantly associated with performance benefits on congruent trials.

The RT model revealed no main or interaction effects with Reward Responsiveness or BAS drive.

Supplementary Table 7

Accuracy model results per experiment

Predictor	Exp.	Estimate	Est. Error	95% CI
**Intercept**	Pilot	2.256	0.094	2.071–2.442
	1	2.588	0.099	2.393–2.782
	2	2.596	0.058	2.484–2.710
	3	2.527	0.100	2.334–2.724
**Group**	Pilot	-0.023	0.085	-0.191–0.144
	1	0.013	0.092	-0.167–0.197
	2	-0.020	0.051	-0.121–0.079
	3	0.003	0.091	-0.175–0.182
**Congruency**	Pilot	0.284	0.035	0.214–0.353
	1	0.360	0.033	0.296–0.425
	2	0.265	0.021	0.224–0.306
	3	0.275	0.028	0.219–0.330
**Transition**	Pilot	0.140	0.022	0.098–0.183
	1	0.155	0.027	0.101–0.208
	2	0.165	0.019	0.129–0.202
	3	0.095	0.024	0.049–0.142
**Task**	Pilot	0.141	0.044	0.055–0.228
	1	0.215	0.043	0.133–0.299
	2	0.281	0.034	0.215–0.349
	3	0.205	0.042	0.124–0.288
**Group*Congruency**	Pilot	-0.007	0.035	-0.075–0.060
	1	0.037	0.031	-0.023–0.098
	2	0.026	0.020	-0.013–0.065
	3	0.053	0.027	0.001–0.105
**Group*Transition**	Pilot	-0.030	0.021	-0.071–0.011
	1	0.023	0.025	-0.026–0.072
	2	-0.020	0.018	-0.054–0.015
	3	-0.001	0.023	-0.045–0.043
**Congruency*Transition**	Pilot	-0.010	0.021	-0.051–0.032
	1	-0.013	0.023	-0.058–0.033
	2	-0.013	0.017	-0.046–0.022
	3	-0.063	0.023	-0.108–-0.017
**Group*Congruency*Transition**	Pilot	-0.030	0.021	-0.070–0.011
	1	-0.026	0.022	-0.069–0.018
	2	0.006	0.016	-0.024–0.037
	3	0.012	0.022	-0.030–0.055

Supplementary Table 8

RT model results per experiment

Predictor	Exp.	Estimate	Est. Error	95% CI
**Intercept**	Pilot	6.963	0.028	6.908–7.016
	1	6.757	0.028	6.702–6.812
	2	6.762	0.016	6.730–6.794
	3	6.246	0.034	6.179–6.314
**Group**	Pilot	-0.021	0.025	-0.069–0.029
	1	-0.006	0.027	-0.058–0.046
	2	-0.001	0.015	-0.031–0.028
	3	0.004	0.035	-0.065–0.074
**Congruency**	Pilot	-0.017	0.002	-0.021–-0.012
	1	-0.020	0.002	-0.025–-0.016
	2	-0.015	0.002	-0.018–-0.012
	3	-0.010	0.003	-0.016–-0.005
**Transition**	Pilot	-0.076	0.004	-0.085–-0.068
	1	-0.073	0.004	-0.081–-0.066
	2	-0.082	0.003	-0.087–-0.076
	3	-0.026	0.003	-0.032–-0.019
**Task**	Pilot	-0.041	0.006	-0.052–-0.030
	1	-0.036	0.005	-0.047–-0.026
	2	-0.064	0.005	-0.073–-0.055
	3	-0.058	0.006	-0.070–-0.046
**Group*Congruency**	Pilot	0.004	0.002	-0.001–0.008
	1	-0.002	0.002	-0.006–0.003
	2	-0.000	0.002	-0.004–0.003
	3	0.008	0.003	0.003–0.014
**Group*Transition**	Pilot	0.000	0.004	-0.007–0.008
	1	-0.003	0.004	-0.010–0.004
	2	0.003	0.003	-0.003–0.009
	3	-0.004	0.003	-0.011–0.002
**Congruency*Transition**	Pilot	0.001	0.002	-0.003–0.005
	1	-0.000	0.002	-0.004–0.004
	2	0.001	0.001	-0.002–0.004
	3	0.001	0.002	-0.004–0.005
**Group*Congruency*Transition**	Pilot	0.000	0.002	-0.004–0.004
	1	-0.001	0.002	-0.005–0.003
	2	-0.001	0.001	-0.004–0.002
	3	0.004	0.002	-0.001–0.009

Supplementary Table 9

Accuracy model results excluding the pilot

Predictor	Estimate	Est. Error	95% CI
Intercept	2.559	0.048	2.464–2.652
Group	-0.000	0.043	-0.085–0.083
Congruency	0.299	0.016	0.268–0.330
Transition	0.139	0.014	0.111–0.166
Experiment 1	0.045	0.064	-0.081–0.171
Experiment 2	0.019	0.058	-0.095–0.131
Task 1	0.244	0.025	0.196–0.292
Group*Congruency	0.038	0.015	0.009–0.067
Group*Transition	0.001	0.013	-0.024–0.027
Congruency*Transition	-0.029	0.012	-0.053–-0.004
Group*Experiment 1	0.015	0.064	-0.112–0.141
Group*Experiment 2	-0.023	0.054	-0.129–0.083
Congruency*Experiment 1	0.063	0.023	0.019–0.108
Congruency*Experiment 2	-0.037	0.019	-0.074–0.001
Transition*Experiment 1	0.027	0.019	-0.012–0.064
Transition*Experiment 2	0.023	0.017	-0.010–0.055
Group*Congruency*Transition	-0.000	0.011	-0.022–0.022
Group1*Congruency*Experiment 1	-0.000	0.022	-0.044–0.044
Group1*Congruency1*Experiment 2	-0.011	0.019	-0.048–0.026
Group*Transition*Experiment 1	0.0212	0.019	-0.016–0.059
Group*Transition*Experiment 2	-0.021	0.017	-0.053–0.012
Congruency*Transition*Experiment 1	0.019	0.017	-0.015–0.052
Congruency*Transition*Experiment 2	0.014	0.014	-0.015–0.042
Group*Congruency*Transition1*Experiment 1	-0.022	0.017	-0.055–0.012
Group*Congruency*Transition1*Experiment 2	0.006	0.014	-0.022–0.034

Supplementary Table 10

RT model results excluding the pilot

Predictor	Estimate	Est. Error	95% CI
Intercept	6.622	0.014	6.594–6.650
Group	0.000	0.013	-0.026–0.026
Congruency	-0.015	0.001	-0.018–-0.013
Transition	-0.061	0.002	-0.065–-0.057
Experiment 1	0.101	0.020	0.062–0.140
Experiment 2	0.115	0.017	0.081–0.149
Task 1	-0.054	0.003	-0.060–-0.048
Group*Congruency	0.001	0.001	-0.001–0.004
Group*Transition	-0.001	0.002	-0.005–0.003
Congruency*Transition	0.001	0.001	-0.001–0.003
Group*Experiment 1	-0.003	0.020	-0.042–0.035
Group*Experiment 2	-0.001	0.016	-0.033–0.031
Congruency*Experiment 1	-0.006	0.002	-0.009–-0.002
Congruency*Experiment 2	-0.001	0.002	-0.004–0.002
Transition*Experiment 1	-0.016	0.003	-0.022–-0.010
Transition*Experiment 2	-0.022	0.003	-0.027–-0.017
Group*Congruency*Transition	0.001	0.001	-0.002–0.003
Group1*Congruency*Experiment 1	-0.003	0.002	-0.007–0.001
Group1*Congruency1*Experiment 2	-0.002	0.002	-0.005–0.001
Group*Transition*Experiment 1	-0.002	0.003	-0.008–0.004
Group*Transition*Experiment 2	0.004	0.003	-0.001–0.009
Congruency*Transition*Experiment 1	-0.001	0.002	-0.004–0.003
Congruency*Transition*Experiment 2	0.001	0.001	-0.002–0.003
Group*Congruency*Transition1*Experiment 1	-0.001	0.002	-0.005–0.002
Group*Congruency*Transition1*Experiment 2	-0.001	0.001	-0.004–0.001

Supplementary Table 11

Accuracy Modelling results of combined experiment analyses including pilot

**Predictor**	**Estimate**	**Est. Error**	**95% CI**
Intercept	2.480	0.045	2.392–2.567
Group	-0.006	0.038	-0.081–0.069
Congruency	0.296	0.014	0.268–0.324
Transition	0.139	0.012	0.116–0.162
Pilot	-0.224	0.071	-0.363–-0.083
Experiment 1	0.116	0.070	-0.023–0.251
Experiment 2	0.097	0.061	-0.021–0.219
Task	0.225	0.023	0.180–0.271
Group*Congruency	0.029	0.014	0.001–0.056
Group*Transition	-0.005	0.011	-0.027–0.016
Congruency*Transition	-0.023	0.011	-0.044–-0.002
Group* Pilot	-0.024	0.069	-0.160–0.114
Group*Experiment 1	0.026	0.070	-0.111–0.163
Group*Experiment 2	-0.016	0.055	-0.124–0.092
Congruency* Pilot	-0.021	0.025	-0.070–0.027
Congruency*Experiment 1	0.070	0.026	0.020–0.120
Congruency*Experiment 2	-0.030	0.021	-0.070–0.011
Transition* Pilot	0.000	0.019	-0.038–0.038
Transition*Experiment 1	0.027	0.020	-0.013–0.067
Transition*Experiment 2	0.022	0.016	-0.010–0.055
Group*Congruency*Transition	-0.007	0.010	-0.027–0.012
Group1*Congruency* Pilot	-0.028	0.025	-0.076–0.021
Group1*Congruency1*Experiment 1	0.010	0.026	-0.040–0.060
Group1*Congruency1*Experiment 2	-0.002	0.021	-0.043–0.038
Group*Transition* Pilot	-0.020	0.019	-0.057–0.018
Group*Transition*Experiment 1	0.027	0.020	-0.012–0.067
Group*Transition*Experiment 2	-0.014	0.016	-0.046–0.018
Congruency*Transition* Pilot	0.013	0.017	-0.021–0.048
Congruency*Transition*Experiment 1	0.015	0.018	-0.021–0.051
Congruency*Transition*Experiment 2	0.009	0.015	-0.020–0.037
Group*Congruency*Transition1*Pilot	-0.022	0.017	-0.056–0.012
Group*Congruency*Transition1*Experiment 1	-0.014	0.018	-0.050–0.022
Group*Congruency*Transition1*Experiment 2	0.013	0.015	-0.016–0.042

Supplementary Table 12

RT Modelling results of combined experiment analyses including pilot

**Predictors**	**Estimate**	**Est. Error**	**95% CI**
Intercept	6.686	0.013	6.661–6.711
Group	-0.006	0.012	-0.030–0.017
Congruency	-0.016	0.001	-0.018–-0.014
Transition	-0.067	0.002	-0.070–-0.063
Pilot	0.185	0.022	0.144–0.227
Experiment 2	0.037	0.022	-0.004–0.080
Experiment 3	0.053	0.018	0.019–0.088
Task	-0.053	0.003	-0.059–-0.046
Group*Congruency	0.002	0.001	-0.000–0.004
Group*Transition	-0.001	0.002	-0.004–0.003
Congruency*Transition	0.001	0.001	-0.001–0.002
Group*Pilot	-0.016	0.021	-0.058–0.025
Group*Experiment 1	-0.000	0.022	-0.041–0.043
Group*Experiment 2	0.004	0.018	-0.032–0.039
Congruency*Pilot	-0.003	0.002	-0.007–0.001
Congruency*Experiment 1	-0.005	0.002	-0.009–-0.001
Congruency*Experiment 2	0.000	0.002	-0.003–0.003
Transition*Pilot	-0.017	0.003	-0.023–-0.010
Transition*Experiment 1	-0.009	0.003	-0.016–-0.003
Transition*Experiment 2	-0.017	0.003	-0.022–-0.012
Group*Congruency*Transition	0.001	0.001	-0.001–0.002
Group1*Congruency*Pilot	0.002	0.002	-0.002–0.006
Group1*Congruency1*Experiment 1	-0.004	0.002	-0.008–0.000
Group1*Congruency1*Experiment 2	-0.003	0.002	-0.006–0.001
Group*Transition*Pilot	0.000	0.003	-0.006–0.007
Group*Transition*Experiment 1	-0.002	0.003	-0.008–0.005
Group*Transition*Experiment 2	0.003	0.003	-0.002–0.009
Congruency*Transition*Pilot	0.000	0.002	-0.003–0.003
Congruency*Transition*Experiment 1	-0.001	0.002	-0.004–0.003
Congruency*Transition*Experiment 2	0.001	0.001	-0.002–0.003
Group*Congruency*Transition1*Pilot	-0.000	0.002	-0.004–0.003
Group*Congruency*Transition1*Experiment 1	-0.001	0.002	-0.005–0.002
Group*Congruency*Transition1*Experiment 2	-0.001	0.001	-0.004–0.001

Supplementary Methods 3

All DDM analyses were performed using R. Evidence accumulation was simulated using the Euler-Maruyama method using the Rcpp package[2-4] and high-performance computing infrastructure to increase speed, as described in Desender, Vermeylen & Verguts[5].

Evidence was accumulated with the following formula

$$delta_{evidence} = evidence + drift * dt + sqrt(dt) * N(0,1) * s$$

accumulating until a given time was reached (shift time), after which the boundary shifted. This timing was variable across congruency levels (drawn from normal distribution) thereby resulting in continuously chaining boundaries. Per participant, we fitted 500 iterations with 20000 simulations. The gamma function of the conflict model was fitted with a gamma function as described in Ulrich et al., (2015)

$$v(t) = C * \beta * e^{-\frac{t}{\tau}} * \left[\frac{t * e}{(\alpha - 1) * \tau}\right]^{\alpha - 1} * \left[\frac{\alpha - 1}{t} - \frac{1}{\tau}\right] + vc$$

$$v(t) = -C * \beta * e^{-\frac{t}{\tau}} * \left[\frac{t * e}{(\alpha - 1) * \tau}\right]^{\alpha - 1} * \left[\frac{\alpha - 1}{t} - \frac{1}{\tau}\right] + vc$$

with C being positive on congruent and negative on incongruent trials.

Supplementary Table 13

Parameter bounds used for DDM modelling

	**v**	**a**	**t**	**st**	**amp**	**tau**	**sshift**	**shift time**	**shift**
DDM	0.00,0.50	-0.05, 0.30	0.20, 0.70	0.00,0.20	-	-	-	-	-
DDM with shift	0.00, 0.50	-0.05, 0.20	0.20, 0.70	0.00,0.20	-	-	0.00,0.40	0.10, 7.00	-0.05, 0.10
DMC	0, 0.50	50,300	200,700	0,200	5, 50	5,250	-	-	-
DMC with shift	0, 0.50	50,200	200,700	0,200	5,50	5,250	0,400	100,700	-50, 100

Note. V: drift rate (separate for congruent and incongruent trials in the DDM models), a: boundary parameter, t: non-decision time, st: variability in non-decision time, amp: peak amplitude of automatic gamma function, tau: latency of automatic gamma function, sshift: variability in time of boundary shift, shift time: time of boundary shift, shift: shift between initial and adapted boundary threshold (separate for congruent and incongruent trials). The shape parameter of the automatic gamma function alpha was fixed at 2, the drift diffusion constant σ fixed at 0.1 in the DDMs and at 4 in the DMCs. We assumed no bias in starting point, and no variability in starting point or drift rate.

Supplementary Methods 4

To assess model fit, we compared correlations between predicted and observed values (accuracy and RT) between the models. For each participant (n=415), we simulated data based on the estimated parameters (10000 trials). For both the observed and simulated data, we then calculated mean correct RT for each RT quintile (quintiles determined separately for each congruency level; see Figure).

Figure

Correlations between predicted and observed values

Standard DDM

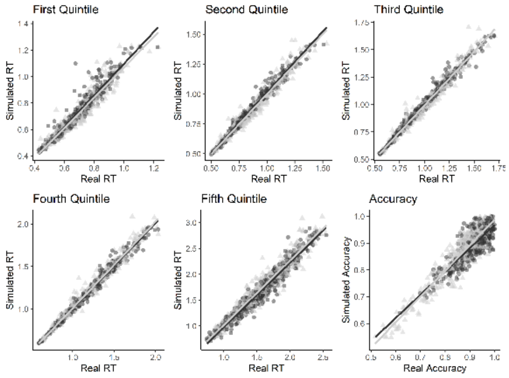

DDM Shift

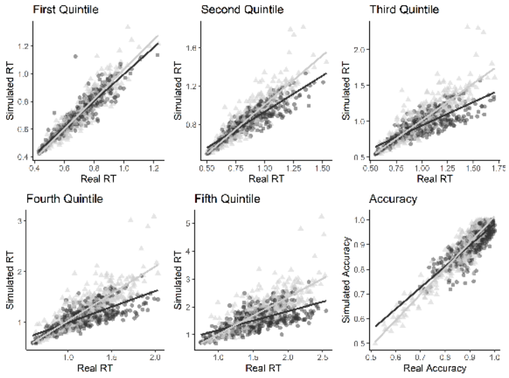

DMC

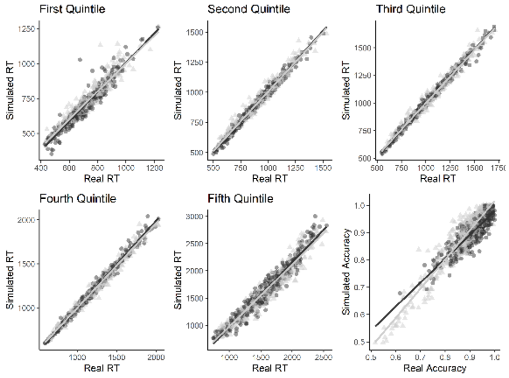

DMC Shift

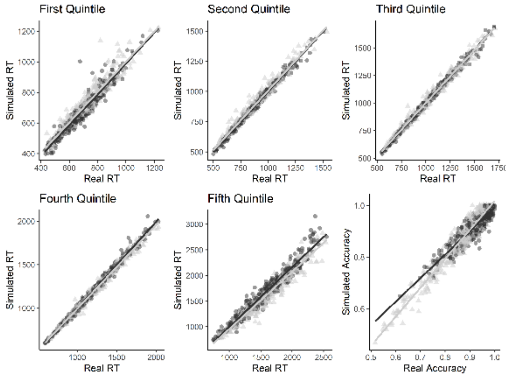

Note. congruent trials are in black, incongruent trials in grey.

Supplementary Table 14

KS statistics and correlations between predicted and observed values per model

Model	Cumulative KS statistic	Pearson's correlations	
		Accuracy	Correct RT
DDM	45.49	0.91(828), *p*<0.001	0.98(4148), *p*<0.001
DDM with shift	37.33	0.92(828), *p*<0.001	0.89(4148), *p*<0.001
DMC	41.43	0.92 (828), *p*<0.001	0.99(4148), *p*<0.001
DMC with shift	37.43	0.95 (828), *p*<0.001	0.99(4148), *p*<0.001

Note. RT correlations did not take clustering within participants into account; correlations are calculated across quintiles and congruency levels.

Supplementary Table 15

Model estimates of different DDMs and t-tests

Model	Parameter	*Congruent group*		*Incongruent group*					
		Mean	***SD***	***Mean***	***SD***	***t*(413)**	***p***	**Cohen's d**	**95 % CI**
DDM	congruent v	0.15	0.05	0.15	0.06	-0.520	0.604	-0.05	-0.24–0.14
	incongruent v	0.12	0.05	0.13	0.05	-0.527	0.599	-0.05	-0.24–0.14
	v difference	0.02	0.04	0.02	0.04	-0.033	0.974	-0.00	-0.20–0.19
	a	0.16	0.03	0.16	0.03	-0.117	0.907	-0.01	-0.20–0.18
	t	0.59	0.12	0.59	0.14	-0.525	0.600	-0.05	-0.24–0.14
	st	0.15	0.06	0.16	0.05	-0.800	0.424	-0.08	-0.27–0.11
DDM shift	congruent v	0.17	0.06	0.17	0.07	0.246	0.806	0.02	-0.17–0.22
	incongruent v	0.13	0.06	0.14	0.07	-1.658	0.098	-0.16	-0.36–0.03
	v difference	0.04	0.05	0.02	0.05	2.426	0.016	0.24	0.04–0.43
	a	0.16	0.03	0.16	0.03	0.966	0.335	0.09	-0.10–0.29
	t	0.57	0.11	0.58	0.12	-0.677	0.499	-0.07	-0.26–0.13
	st	0.16	0.05	0.16	0.04	-1.262	0.208	-0.12	-0.32–0.07
	sshift	0.18	0.12	0.17	0.11	0.445	0.657	0.04	-0.15–0.24
	shift time	0.39	0.20	0.36	0.21	1.348	0.178	0.13	-0.06–0.32
	congruent shift	0.01	0.04	0.01	0.04	0.264	0.792	0.03	-0.17–0.22
	incongruent shift	0.00	0.03	0.01	0.04	-2.291	0.023	-0.23	-0.42–-0.03

	shift difference	0.01	0.03	0.00	0.03	3.011	0.003	0.30	0.10–0.49
DMC	v	0.19	0.06	0.19	0.06	-0.684	0.495	-0.07	-0.26–0.13
	a	102.25	28.92	101.46	29.04	0.276	0.783	0.03	-0.17–0.22
	t	604.06	128.44	621.11	145.48	-1.267	0.206	-0.12	-0.32–0.07
	st	106.19	62.78	112.32	66.97	-0.962	0.336	-0.09	-0.29–0.10
	amp	22.10	14.03	21.19	13.87	0.664	0.507	0.07	-0.13–0.26
	tau	110.71	98.07	118.05	100.97	-0.751	0.453	-0.07	-0.27–0.12
DMC shift	v	0.20	0.07	0.20	0.07	-0.483	0.629	-0.05	-0.24–0.15
	a	101.66	32.01	104.37	32.28	-0.857	0.392	-0.08	-0.28–0.11
	t	608.30	125.82	610.46	139.51	-0.166	0.869	-0.02	-0.21–0.18
	st	109.92	58.56	111.35	59.35	-0.246	0.806	-0.02	-0.22–0.17
	amp	18.14	10.81	19.31	11.05	1.091	0.276	-0.11	-0.30–0.09
	tau	118.51	91.79	114.39	89.06	0.465	0.643	0.05	-0.15–0.24
	sshift	201.99	117.27	205.14	119.62	-0.270	0.787	-0.03	-0.22–0.17
	shift time	409.99	178.02	430.95	165.53	-1.241	0.215	-0.12	-0.31–0.07
	congruent shift	13.40	37.55	8.42	36.34	1.373	0.171	0.13	-0.06–0.33
	incongruent shift	1.98	40.65	2.30	39.04	-0.083	0.934	-0.00	-0.20–0.18
	shift difference	11.42	29.35	6.11	27.89	1.887	0.060	0.19	-0.01–0.38

Note. Where variance differed between, groups, Welch (or Satterthwaite) approximation to the degrees of freedom was used.

Supplementary Table 16

Accuracy results from second half of Experiment 3

	**Reward.**	**Estimate**	**Est. Error**	**95% CI**
Intercept	A	2.972	0.069	2.837–3.109
	B	2.906	0.068	2.772–3.041
Group	A	-0.006	0.063	-0.129–0.115
	B	-0.018	0.061	-0.137–0.101
Congruency	A	0.406	0.028	0.351–0.462
	B	0.393	0.027	0.339–0.446
Transition	A	0.127	0.025	0.078–0.176
	B	0.138	0.024	0.091–0.184
Task	A	0.213	0.039	0.137–0.289
	B	0.218	0.037	0.146–0.290
Group*Congruency	A	0.103	0.025	0.054–0.153
	B	0.026	0.025	-0.024–0.076
Group*Transition	A	-0.047	0.023	-0.092–-0.002
	B	-0.061	0.021	-0.103–-0.019
Congruency*Transition	A	-0.015	0.024	-0.063–0.032
	B	-0.029	0.022	-0.074–0.014
Group*Congruency*Transition	A	-0.025	0.023	-0.070–0.019
	B	-0.010	0.020	-0.049–0.030

Note: A: Only previously rewarded stimuli of reward condition. B: only non-previously rewarded stimuli of reward condition

Supplementary Table 17

RT results from second half of Experiment 3

	Reward.	Estimate	Est. Error	95% CI
Intercept	A	6.706	0.018	6.672–6.742
	B	6.714	0.018	6.679–6.750
Group	A	-0.011	0.016	-0.043–0.019
	B	-0.011	0.016	-0.043–0.021
Congruency	A	-0.025	0.002	-0.029–-0.021
	B	-0.024	0.002	-0.028–-0.020
Transition	A	-0.066	0.003	-0.072–-0.060
	B	-0.064	0.003	-0.070–-0.058
Task	A	-0.048	0.005	-0.057–-0.038
	B	-0.047	0.005	-0.057–-0.038
Group*Congruency	A	-0.004	0.002	-0.008–-0.001
	B	-0.002	0.002	-0.006–0.002
Group*Transition	A	-0.000	0.003	-0.006–0.006
	B	-0.002	0.003	-0.008–0.004
Congruency*Transition	A	0.003	0.002	-0.000–0.006
	B	0.001	0.001	-0.001–0.004
Group*Congruency*Transition	A	-0.002	0.002	-0.005–0.001
	B	-0.000	0.001	-0.003–0.003

Note. A: Only previously rewarded stimuli of reward condition. B: only non-previously rewarded stimuli of reward condition

Supplementary Table 18

Accuracy results including similarity factor for analyses on second half in Experiment 3

	**Estimate**	**Est. Error**	**95% CI**
Intercept	2.995	0.072	2.854–3.139
Group	-0.015	0.066	-0.145–0.114
Congruency	0.421	0.029	0.364–0.479
Transition	0.130	0.025	0.082–0.178
Task	0.231	0.041	0.150–0.312
Same vs. Different	-0.210	0.027	-0.263–-0.159
Group*Congruency	0.101	0.027	0.049–0.154
Group*Transition	-0.042	0.023	-0.086–0.004
Group*Task	0.049	0.030	-0.010–0.108
Group*Same vs. Different	0.015	0.024	-0.032–0.061
Congruency*Transition	-0.013	0.024	-0.060–0.034
Congruency*Task	0.106	0.025	0.057–0.155
Congruency*Same vs. Different	0.034	0.026	-0.018–0.083
Group*Congruency*Transition	-0.021	0.023	-0.065–0.024
Group*Congruency*Task	-0.017	0.023	-0.062–0.029
Group*Congruency*Same vs. Different	-0.017	0.023	-0.063–0.028

Supplementary Table 19

RT results including similarity factor for analyses on second half in Experiment 3

	**Estimate**	**Est. Error**	**95% CI**
Intercept	6.715	0.018	6.680–6.750
Group	-0.013	0.016	-0.045–0.020
Congruency	-0.025	0.002	-0.029–-0.021
Transition	-0.065	0.003	-0.071–-0.059
Task	-0.047	0.005	-0.057–-0.038
Same vs. Different	0.022	0.002	0.019–0.026
Group*Congruency	-0.004	0.002	-0.008–-0.001
Group*Transition	-0.000	0.003	-0.006–0.006
Group*Task	0.003	0.003	-0.004–0.010
Group*Same vs. Different	-0.002	0.002	-0.006–0.001
Congruency*Transition	0.003	0.002	0.000–0.006
Congruency*Task	0.002	0.002	-0.002–0.005
Congruency*Same vs. Different	-0.004	0.002	-0.007–-0.001
Group*Congruency*Transition	-0.002	0.002	-0.005–0.001
Group*Congruency*Task	-0.001	0.002	-0.004–0.003
Group*Congruency*Same vs. Different	0.001	0.002	-0.002–0.004

Supplementary References

1. Carver, C. S., & White, T. L. (1994). Behavioral inhibition, behavioral activation, and affective responses to impending reward and punishment: The BIS/BAS Scales. *Journal of Personality and Social Psychology*, *67*(2), Article 2. https://doi.org/10.1037/0022-3514.67.2.319
2. Eddelbuettel, D. (2013). *Seamless R and C++ Integration with Rcpp*. Springer New York. https://doi.org/10.1007/978-1-4614-6868-4
3. Eddelbuettel, D., & Balamuta, J. J. (2018). Extending *R* with C++: A Brief Introduction to Rcpp. *The American Statistician*, *72*(1), 28–36. https://doi.org/10.1080/00031305.2017.1375990
4. Eddelbuettel, D., & François, R. (2011). Rcpp: Seamless *R* and *C++* Integration. *Journal of Statistical Software*, *40*(8). https://doi.org/10.18637/jss.v040.i08
5. Desender, K., Vermeylen, L., & Verguts, T. (2022). Dynamic influences on static measures of metacognition. *Nature Communications*, *13*(1), 4208. https://doi.org/10.1038/s41467-022-31727-0
