# Supplementary material for: Reinforcement learning of adaptive control strategies
Source: Commun Psychol. 2024 Jan 12;2:8. doi: 10.1038/s44271-024-00055-y (PMC11332247; doi:10.1038/s44271-024-00055-y)
Supplement: Supplementary file 3 — Reporting Summary [file 44271_2024_55_MOESM3_ESM.pdf]

nature portfolio

Corresponding author(s): Leslie Held

Last updated by author(s): Dec 7, 2023

Reporting Summary

Nature Portfolio wishes to improve the reproducibility of the work that we publish. This form provides structure for consistency and transparency in reporting. For further information on Nature Portfolio policies, see our Editorial Policies and the Editorial Policy Checklist.

Statistics

For all statistical analyses, confirm that the following items are present in the figure legend, table legend, main text, or Methods section.

n/a	Confirmed	
☐	☒	The exact sample size (*n*) for each experimental group/condition, given as a discrete number and unit of measurement
☐	☒	A statement on whether measurements were taken from distinct samples or whether the same sample was measured repeatedly
☐	☒	The statistical test(s) used AND whether they are one- or two-sided<br>*Only common tests should be described solely by name; describe more complex techniques in the Methods section.*
☐	☒	A description of all covariates tested
☐	☒	A description of any assumptions or corrections, such as tests of normality and adjustment for multiple comparisons
☐	☒	A full description of the statistical parameters including central tendency (e.g. means) or other basic estimates (e.g. regression coefficient) AND variation (e.g. standard deviation) or associated estimates of uncertainty (e.g. confidence intervals)
☐	☒	For null hypothesis testing, the test statistic (e.g. *F*, *t*, *r*) with confidence intervals, effect sizes, degrees of freedom and *P* value noted<br>*Give P values as exact values whenever suitable.*
☐	☒	For Bayesian analysis, information on the choice of priors and Markov chain Monte Carlo settings
☒	☐	For hierarchical and complex designs, identification of the appropriate level for tests and full reporting of outcomes
☐	☒	Estimates of effect sizes (e.g. Cohen's *d*, Pearson's *r*), indicating how they were calculated

Our web collection on statistics for biologists contains articles on many of the points above.

Software and code

Policy information about availability of computer code

Data collection	jspsych-6.0.5 was used to program the experiment.
Data analysis	brms (2.19.0) and RoBMA (2.3.1) were used for main statistical analyses.

For manuscripts utilizing custom algorithms or software that are central to the research but not yet described in published literature, software must be made available to editors and reviewers. We strongly encourage code deposition in a community repository (e.g. GitHub). See the Nature Portfolio guidelines for submitting code & software for further information.

Data

Policy information about availability of data

All manuscripts must include a data availability statement. This statement should provide the following information, where applicable:

- Accession codes, unique identifiers, or web links for publicly available datasets
- A description of any restrictions on data availability
- For clinical datasets or third party data, please ensure that the statement adheres to our policy

All data and materials is made available on OSF (https://osf.io/qdk5t/).

Human research participants

Policy information about studies involving human research participants and Sex and Gender in Research.

Reporting on sex and gender	Gender (M, F, X) was collected as default demographic information, along with age and handedness. We did not perform analyses on these data as we had no reason to assume differences or hypotheses on gender differences based on previous research.
Population characteristics	All participants were between 18 and 35 years old and native English speakers.
Recruitment	Participants were recruited via Prolific (https://www.prolific.co/).
Ethics oversight	The study was approved by the Ghent University Faculty of Psychology and Educational Sciences Ethical Commission.

Note that full information on the approval of the study protocol must also be provided in the manuscript.

Field-specific reporting

Please select the one below that is the best fit for your research. If you are not sure, read the appropriate sections before making your selection.

☐ Life sciences ☒ Behavioural & social sciences ☐ Ecological, evolutionary & environmental sciences

For a reference copy of the document with all sections, see nature.com/documents/nr-reporting-summary-flat.pdf

Behavioural & social sciences study design

All studies must disclose on these points even when the disclosure is negative.

Study description	The study was experimental (between-subjects/quantitative).
Research sample	The sample consisted of native English speakers with a Prolific account (316 females, 96 males, 3 other, mean age (floored) 22 (SD=5), 90% right-handed).
Sampling strategy	The sampling procedure was random. We used a preregistered default sample size for between-subject studies per study (50 per group) which exceeds the sample size of previous studies testing similar effects.
Data collection	Participants performed the experiment at home on their own computers.
Timing	All data was collected in 2021-2022.
Data exclusions	15 participants were excluded based on preregistered exclusion criteria (e.g., accuracy below 60% per task). Additional participants were excluded based on obvious attention failures (e.g., significant number of missed trials in a row).
Non-participation	N/A
Randomization	Group allocation was random.

Reporting for specific materials, systems and methods

We require information from authors about some types of materials, experimental systems and methods used in many studies. Here, indicate whether each material, system or method listed is relevant to your study. If you are not sure if a list item applies to your research, read the appropriate section before selecting a response.

Materials & experimental systems

n/a	Involved in the study
☒	☐ Antibodies
☒	☐ Eukaryotic cell lines
☒	☐ Palaeontology and archaeology
☒	☐ Animals and other organisms
☒	☐ Clinical data
☒	☐ Dual use research of concern

Methods

n/a	Involved in the study
☒	☐ ChIP-seq
☒	☐ Flow cytometry
☒	☐ MRI-based neuroimaging
